# Evaluation and Adaptation of a Laboratory-Based cDNA Library Preparation Protocol for Retrospective Sequencing of Archived MicroRNAs from up to 35-Year-Old Clinical FFPE Specimens

**DOI:** 10.3390/ijms18030627

**Published:** 2017-03-14

**Authors:** Olivier Loudig, Tao Wang, Kenny Ye, Juan Lin, Yihong Wang, Andrew Ramnauth, Christina Liu, Azadeh Stark, Dhananjay Chitale, Robert Greenlee, Deborah Multerer, Stacey Honda, Yihe Daida, Heather Spencer Feigelson, Andrew Glass, Fergus J. Couch, Thomas Rohan, Iddo Z. Ben-Dov

**Affiliations:** 1 Department of Epidemiology and Population Health, Albert Einstein College of Medicine, Bronx, NY 10461, USA; tao.wang@einstein.yu.edu (T.W.); kenny.ye@einstein.yu.edu (K.Y.); Juan.lin@einstein.yu.edu (J.L.); andrew.ramnauth12@myhunter.cuny.edu (A.R.); christina.liu@einstein.yu.edu (C.L.); thomas.rohan@einstein.yu.edu (T.R.); 2 Department of Pathology, Rhode Island Hospital, Providence, RI 02903, USA; ywang6@Lifespan.org; 3 Department of Pathology and Breast Oncology Program, Henry Ford Health System, Detroit, MI 48202, USA; ASTARK1@hfhs.org (A.S.); dchital1@hfhs.org (D.C.); 4 Center for Clinical Epidemiology and Population Health, Marshfield Clinic Research Foundation, Marshfield, WI 54449, USA; greenlee.robert@mcrf.mfldclin.edu (R.G.); multerer.deborah@mcrf.mfldclin.edu (D.M.); 5 Department of Pathology, Center for Health Research, Kaiser Permanente, 3288 Moanalua Road, Honolulu, HI 96819, USA; Stacey.Honda@kp.org (S.H.); Yihe.G.Daida@kp.org (Y.D.); 6 Center for Excellence in Cancer and Genomics, Kaiser Permanente Colorado, Denver, CO 80237, USA; heather.s.feigelson@kp.org; 7 Center for Health Research, Kaiser Permanente Northwest, Portland, OR 97227, USA; andy_5241@msn.com; 8 Health Sciences Research, Mayo Clinic, Rochester, NY 55902, USA; couch.fergus@mayo.edu; 9 Montefiore Medical Center, Bronx, NY 10467, USA; 10 Laboratory of Medical Transcriptomics, Hadassah-Hebrew University Medical Center, Jerusalem 91120, Israel; Iddo@hadassah.org.il

**Keywords:** microRNAs, next-generation sequencing, cDNA library preparation, formalin-fixed paraffin-embedded, ductal carcinoma in situ, invasive breast cancer

## Abstract

Formalin-fixed paraffin-embedded (FFPE) specimens, when used in conjunction with patient clinical data history, represent an invaluable resource for molecular studies of cancer. Even though nucleic acids extracted from archived FFPE tissues are degraded, their molecular analysis has become possible. In this study, we optimized a laboratory-based next-generation sequencing barcoded cDNA library preparation protocol for analysis of small RNAs recovered from archived FFPE tissues. Using matched fresh and FFPE specimens, we evaluated the robustness and reproducibility of our optimized approach, as well as its applicability to archived clinical specimens stored for up to 35 years. We then evaluated this cDNA library preparation protocol by performing a miRNA expression analysis of archived breast ductal carcinoma in situ (DCIS) specimens, selected for their relation to the risk of subsequent breast cancer development and obtained from six different institutions. Our analyses identified six miRNAs (miR-29a, miR-221, miR-375, miR-184, miR-363, miR-455-5p) differentially expressed between DCIS lesions from women who subsequently developed an invasive breast cancer (cases) and women who did not develop invasive breast cancer within the same time interval (control). Our thorough evaluation and application of this laboratory-based miRNA sequencing analysis indicates that the preparation of small RNA cDNA libraries can reliably be performed on older, archived, clinically-classified specimens.

## 1. Introduction

MicroRNAs (miRNAs) are short (~22-nucleotide) regulatory non-coding single-stranded RNA molecules that can regulate gene expression post-transcriptionally by a variety of mechanisms, which include binding to the 3′ untranslated regions of an mRNA target to downregulate translation, direct degradation or destabilization of the transcript [1,2]. miRNAs are master regulators, which are involved in virtually all biological processes [3]. Dysregulation of their expression has been associated with many diseases and suggested to play important roles in the initiation and progression of a variety of human malignancies [4,5,6,7,8]. MiRNA expression profiling of tumor tissues has been useful for the classification and diagnosis of several human malignancies and has revealed that their expression can be upregulated or downregulated, when compared to normal tissues [5,7,9,10,11].

Gene-expression studies using formalin-fixed paraffin-embedded (FFPE) tissues have demonstrated that degraded nucleic acids can be successfully analyzed [12,13,14]. Using total RNA extracted from FFPE specimens, several studies have demonstrated that miRNAs are preserved in archival tissues and can be reliably quantified using high-throughput technologies [13,15,16,17,18]. The miRNA expression profiling methods that have been used include real-time quantitative PCR (single assays and PCR arrays), microarrays or bead arrays (including NanoString arrays) and, more recently, next-generation sequencing (NGS) [15,16,17,18,19,20,21,22]. Most of the NGS studies performed on archived miRNAs have tested the use of commercial kits.

Aware of the high cost of commercial kits for preparation of cDNA libraries, especially for large-scale studies, we sought to establish a cost-effective and robust laboratory-based cDNA library preparation procedure, compatible with our optimized simultaneous RNA-DNA extraction procedure from FFPE tissues [23]. Considering the limited amount of starting material recovered from archived clinical specimens, we sought to use a method that would allow us to decrease the FFPE RNA input, without compromising data output. For this purpose, we tested and optimized the barcoded cDNA library preparation method and reagents developed by Hafner et al. [24], which had initially been designed to work with 2 μg of total fresh RNA, per barcoded specimen. We selected this protocol because it provides the most adaptable and efficient approach for working with low input amounts of degraded FFPE-RNA, as after individual ligations of 18 RNA samples with 18 different 3′ barcoded adapters, the barcoded RNA samples are pooled together to collectively undergo all subsequent steps of the protocol (carrier effect). Additionally, to render this approach applicable to all molecular biology laboratories, we replaced γ32-ATP-labeled size markers by size markers visualized with SYBR^®^ Gold dye. We also adjusted the concentration of the control spike primers, introduced during initial 3′ ligations of barcoded adapters, to minimize their output during PCR amplification of low input FFPE RNA material. We compared miRNA expression profiles from matched frozen and FFPE tissues for validation of our optimized protocol and tested its applicability with older archived FFPE specimens (up to 35-year-old tissue). Finally, using archived breast ductal carcinoma in situ (DCIS) specimens, for the first time in a prospective setting, we explored the applicability of our approach with clinical specimens obtained from different institutions, for detection of miRNA expression changes correlated with breast cancer development, and used quantitative RT-PCR (qPCR) to evaluate data obtained by our optimized approach.

## 2. Results

### 2.1. Optimized cDNA Library Preparation Protocol for FFPE Small-RNA Sequencing

**Figure 1:**
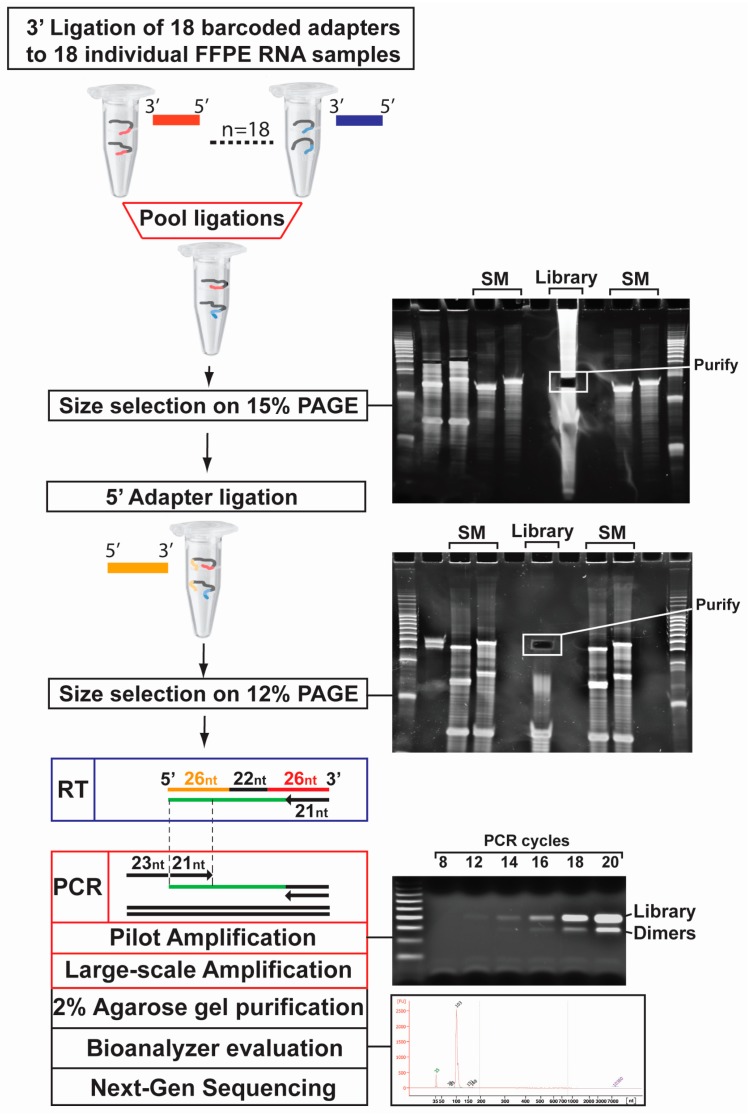
Illustrated representation of the optimized barcoded cDNA library preparation procedure. Total RNA from formalin-fixed paraffin-embedded (FFPE) specimens (*n* = 18), are ligated with 3′ adapters (*n* = 18), purified on a 15% PAGE gel (using size markers (SM) RNA oligonucleotides) and excised after SYBR^®^ gold staining of the gel. The combined purified products are ligated with a 5′ adapter, run on a 12% PAGE gel, excised and purified. The dual-adapter ligated small-RNA products are reverse transcribed, subjected to a pilot PCR for identification of adequate amplification cycle. A large-scale PCR is performed, and the products are separated on a 2% agarose gel, where the upper band (cDNA library) is purified and subjected to sequencing on an Illumina HiSeq-2500 sequencer.

Considering the robustness of the cDNA library preparation protocol developed by Hafner et al. [24] and its versatility for laboratory use, compared to commercial kits, we sought to optimize it for the analysis of degraded FFPE RNA (100 ng per sample). For practicality, we ran size marker oligonucleotides (19 and 24 nt; SM) in separate wells from the FFPE libraries and used them as guides during excision, after staining with SYBR^®^ gold dye (see Figure 1; see the size marker or SM above gel) lanes on both polyacrylamide gels (PAGE)). Following PAGE purification, after 3′ barcoded adapter ligations, all 18 RNA samples were pooled together (Figure 1; see “Library” on 15% PAGE gel) and a single 5′ adapter ligation was set up (Figure 1; see “Library” on 12% PAGE gel). Subsequently, a pilot PCR reaction was set up using the reverse transcribed ligated small-RNA products (Figure 1; see lower gel, 8, 12, 14, 16, 18, 20 cycles) to identify the optimal amplification cycle (generally around 13–15 cycles) for large-scale PCR amplification (six individual reactions of 100 μL). The large-scale PCR amplification generated two bands, one running at ~100 bp and representing our barcoded libraries and one at ~80 bp representing 5′–3′ adapter dimers (Figure 1; see lower gel). After library purification, we used a Bioanalyzer to evaluate library size and purity, prior to sequencing on an Illumina HiSeq 2500.

### 2.2. Evaluation of the cDNA Library Preparation Protocol on FFPE RNA Specimens

**Figure 2:**
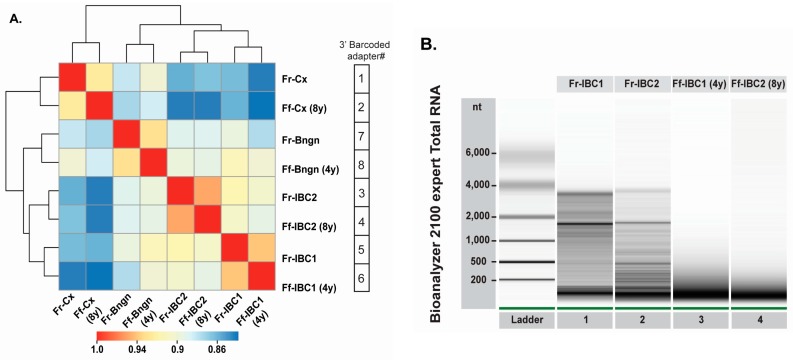

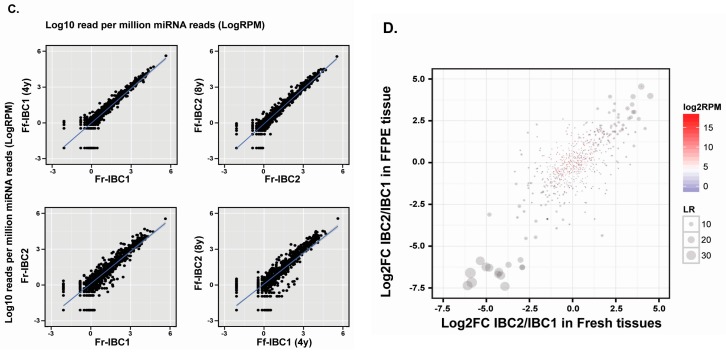
Evaluation of the optimized barcoded cDNA library preparation protocol with matched frozen and FFPE RNA samples. (**A**) Distance mapping of miRNA sequencing expression using the Euclidean distance metric for four pairs of matched frozen and FFPE specimens. Matched frozen (Fr-Cx) and eight-year-old FFPE (Ff-Cx) cervix tissue; matched frozen (Fr-Bngn) and four-year-old FFPE (Ff-Bngn) benign breast tissue; matched frozen (Fr-IBC1) and four-year-old FFPE (Ff-IBC1) invasive ductal carcinoma breast tissue #1; and matched frozen (Fr-IBC1) and eight-year-old FFPE (Ff-IBC2) invasive ductal carcinoma breast tissue #2 were analyzed; (**B**) RNA quality evaluation on a Bioanalyzer total RNA nano chip of matched frozen (Lanes 1 and 2) and FFPE (Lanes 3 and 4) IBC tissue pairs; (**C**) Scatter plot comparisons between matched frozen and FFPE IBC Tissues 1 and 2 (top right and left plots); (**D**) Correlation between IBC2/IBC1 fold-change in matched FFPE and frozen specimens, represented by abundance of miRNAs (log count per million (CPM), red to blue color) and significance of the expression difference between the two tissue pairs (Likelihood Ratio, values above 10 being significant (*p*-value < 0.05)), displaying differentially-expressed miRNAs between two breast tumors (invasive ductal carcinomas).

To determine the applicability of the cDNA library preparation protocol for the analysis of archived FFPE RNA specimens, we prepared our first libraries using the unmodified published procedure from Hafner et al. [24], In this experiment, we used twenty RNA samples (each 1 μg), which included four pairs of matched frozen and FFPE human specimens, for evaluation as controls for reproducibility (Figure 2A). A heat map analysis of the miRNA expression profiles (Figure 2A) displayed that matched fresh and FFPE samples clustered together, with correlation coefficients above 0.93. In Figure S1 [25,26,27,28], we also evaluated the detection of piwiRNAs and transposons in some of the matched frozen and FFPE specimens. In Figure 2A, the unsupervised clustering distributed tissues based on their origin and pathology, with the two sets of human invasive breast cancer specimens (Figure 2A, IBC1 and IBC2) closer to each other. RNA quality measures for fresh and FFPE RNA of the two human breast tumor tissues (Figure 2B) indicated the presence of 18 s and 28 s rRNA in frozen RNA samples (Figure 2B, Lanes 1 and 2), but revealed extensive RNA degradation in the corresponding FFPE RNA samples (Figure 2B, Lanes 3 and 4; RNA Integrity Number (RIN) of 2.1 and 2.4, respectively), with minimal difference between four-year-old and eight-year-old FFPE RNAs. Considering the close phylogeny between the two human IBC specimens (invasive ductal carcinomas), we sought to determine if miRNA expression differences between the two frozen RNA specimens (Fr-IBC1 and Fr-IBC2) could also be detected in their corresponding matched FFPE specimens. Our measures indicate that the correlation between matched frozen and FFPE specimens (Figure 2C, top two panels) was noticeably higher than those for the inter-sample (IBC1 vs. IBC2) comparisons (Figure 2C, lower right panel). By plotting the IBC2/IBC1 miRNA expression ratios from the FFPE tissues (Figure 2D, *Y* axis) compared to miRNA expression ratios from matched frozen tissues (Figure 2D, *X* axis), we show that the detection of miRNA expression in FFPE tissues is a robust surrogate for expression differences identified in frozen tissues (Figure 2D).

### 2.3. Optimal Input of FFPE RNA for Sequencing of Human Archived Tissues

**Figure 3:**
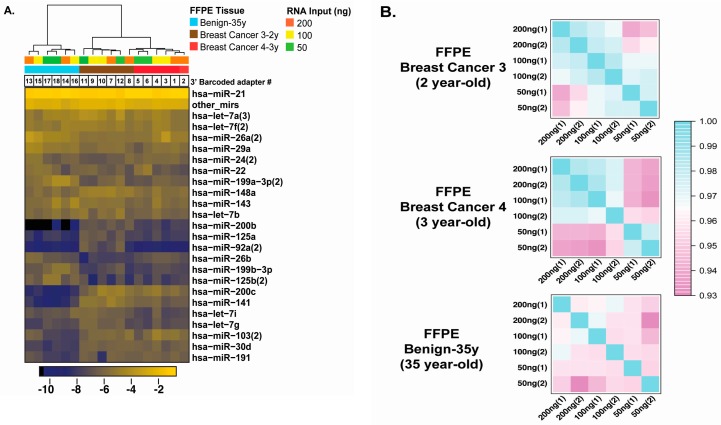

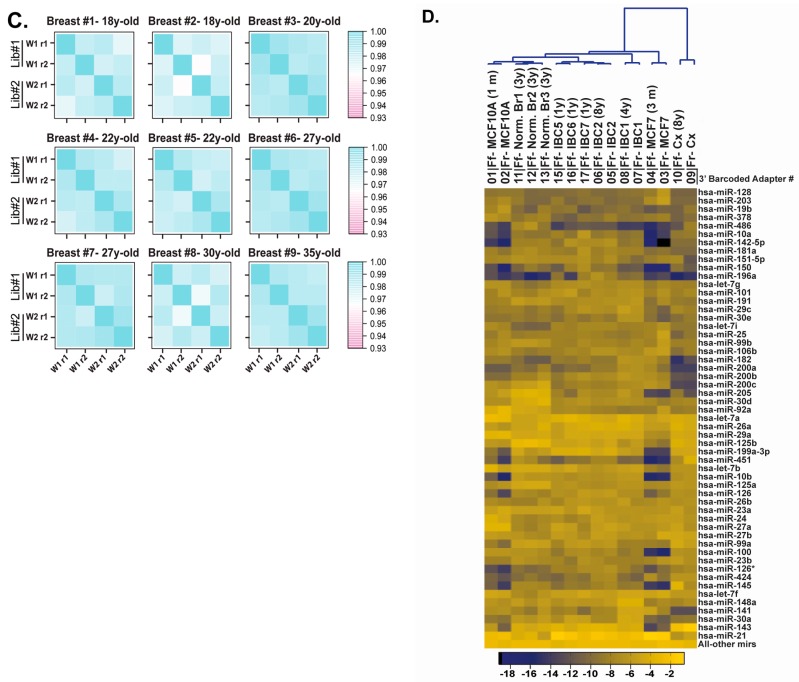
Evaluation of the reproducibility of the barcoded cDNA library preparation procedure. (**A**) Unsupervised clustering of miRNA expression from two-year-old breast cancer #3, three-year-old breast cancer #4 and the 35-year-old benign breast tissue specimens (200, 100 and 50 ng total RNA in duplicates for each specimen); (**B**) Correlation matrices displaying reproducibility between repeats and between different RNA amounts for all three specimens; (**C**) Correlation matrices displaying reproducibility for nine FFPE RNA benign breast tissue specimens. Duplicate measures were performed within the same library (Repeat 1 (r1) and repeat 2 (r2) and between libraries (Lib#1 and Lib#2) prepared at week 1 (w1) and week 2 (w2)) for 18-, 20-, 22-, 27-, 30-, 35-year-old tissues; (**D**) Unsupervised clustering of miRNA expression data from five pairs of matched fresh or frozen and FFPE specimens and six FFPE specimens. The matched pairs include fresh and one-month-old MCF10A FFPE RNA, fresh and three-month old MCF7 FFPE RNA, frozen and four-year-old invasive breast Tumor 1 (IBC1) FFPE RNA, frozen and eight-year-old invasive breast Tumor 2 (IBC2) FFPE RNA, frozen and eight-year-old normal cervix FFPE RNA. The FFPE specimens include three distinct three-year-old normal breast FFPE RNAs (from three different individuals; Formalin-fixed normal breast Ff-Norm-Br#1, #2, and #3) and three distinct one-year-old invasive breast cancer FFPE specimens from three different individuals (Formalin-fixed Invasive Breast Cancer Ff-IBC#3, #4, and #5). The top 75% miRNAs detected by sequencing are represented.

Considering that human archived specimens and specific cell populations within such specimens are generally limited, we sought to determine the minimal RNA input for the application of our optimized cDNA library preparation (Figure 3A). We prepared small-RNA libraries using 200, 100 and 50 ng of FFPE RNA from archived 35-year-old human benign breast tissue, and two-year-old and three-year-old human breast tumor tissues. As there were no matched fresh or frozen total RNA for these samples, we duplicated our measures (200, 100 and 50 ng for all three tissues) to evaluate reproducibility and analyzed the resulting 18 RNA samples (with 18 unique 3′ barcoded adapters) in a single sequencing lane on an Illumina HiSeq 2500. Unsupervised clustering of the 18 libraries (top 24 miRNAs), in the three different tissues (see blue, brown and red), separately clustered the two tumor tissues from the benign breast tissue. To examine RNA input limitations, we evaluated the correlation coefficients between duplicated libraries in individual correlation matrices, for each specimen (Figure 3B). The two human breast cancer specimens displayed a decrease in the correlation coefficients with 50 ng of total FFPE RNA, when compared to 200 and 100 ng, which displayed similar coefficients (Figure 3B, top two panels). Correlation coefficients between replicates for the 35-year-old FFPE benign breast RNA sample were lower than those for two- and three-year-old FFPE tumor tissues (Figure 3B, lower panel). Based on these results, we selected 100 ng of total RNA for subsequent cDNA library preparations (as shown in Figure 1). We then evaluated the reproducibility of the procedure, with 100 ng of FFPE RNA, within an interval of two weeks. Using nine older FFPE specimens, ranging between 18 and 35 years of storage, we compared reproducibility between repeats within a library (Figure 3C; library#1 (lib#1) repeat 1 (r1) and repeat #2 (r2)) and between duplicated libraries prepared on week 1 (w1) and week 2 (w2) (Figure 3C; Library#1 versus Library#2). The correlation matrices indicate high correlation coefficients between repeated libraries within the same week and between different weeks, with r > 0.96, regardless of the age of the specimens. As our final test, we sought to validate our optimized approach using matched fresh-frozen and FFPE specimens and un-matched FFPE samples in a single library, with 100 ng of total RNA per specimen, including specimens we had tested in our first experiment (Figure 2A, IBC1, IBC2 and cervix tissue (Cx)). The unsupervised clustering of the top 54 miRNAs, representing 75% of miRNA sequencing reads, clustered the five individual matched fresh/frozen and FFPE sample pairs correctly. The tumor tissues clustered together (IBC 1, 2, 5, 6, 7), while normal breast tissues 1, 2 and 3 (See norm-Br1, 2, 3) and non-tumorigenic MCF10A samples clustered together. These results indicate that our optimized cDNA library procedure, using 100 ng of total FFPE RNA, can reproducibly capture miRNA expression data relevant to the fresh-frozen tissue/cell line of origin and that it is highly reproducible with older FFPE specimens.

### 2.4. Evaluation of Our Optimized cDNA Library Preparation for the Analysis of Archived DCIS Specimens

Considering that FFPE specimens associated with clinical history can provide valuable information on the molecular dynamics of a disease, we chose to evaluate our optimized procedure with breast ductal carcinoma in situ (DCIS) specimens. In our analysis, we compared the miRNA sequencing profiles of DCIS specimens from women who developed subsequent invasive cancer (cases) to those from women who did not develop invasive breast cancer (controls), within the same time interval. Details of the study specimens are listed in Table 1, including the duration of storage (3–28 years, with a median of 11.5 years), which was lower than the age of the oldest specimen tested during the optimization phase of this project (see Figure 3C, Breast#9 with 35 years of storage) and time to development of IBC (8 months–22 years, with a median of 58 months or ~5 years) after the diagnosis of DCIS.

**Figure 4:**
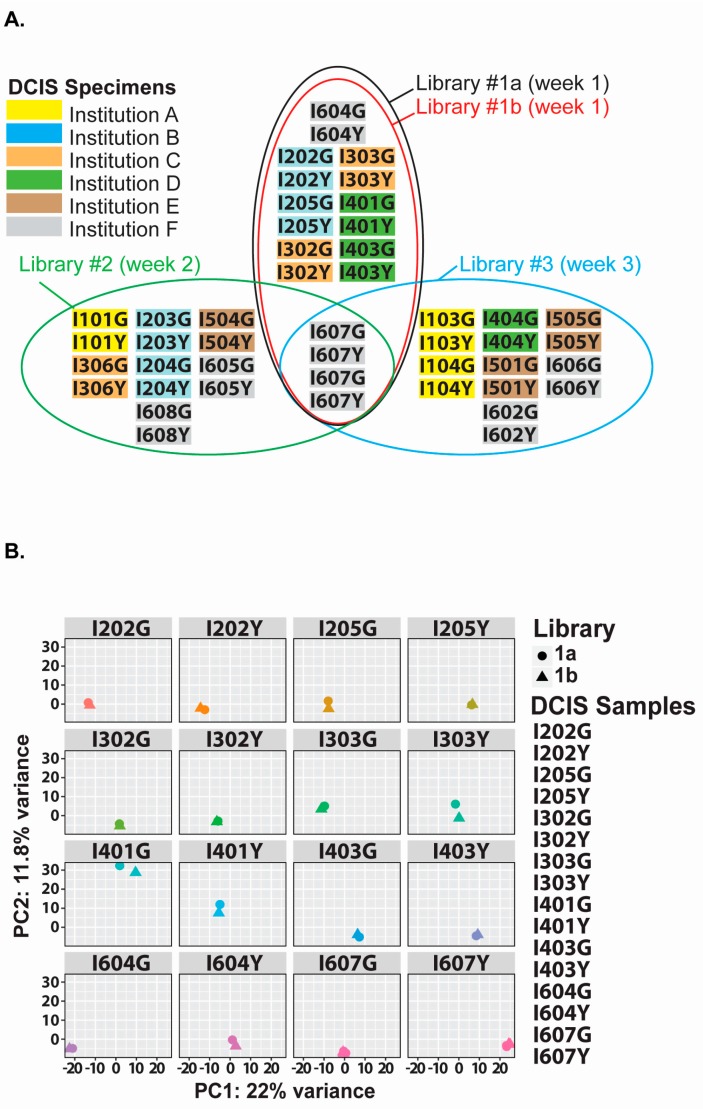

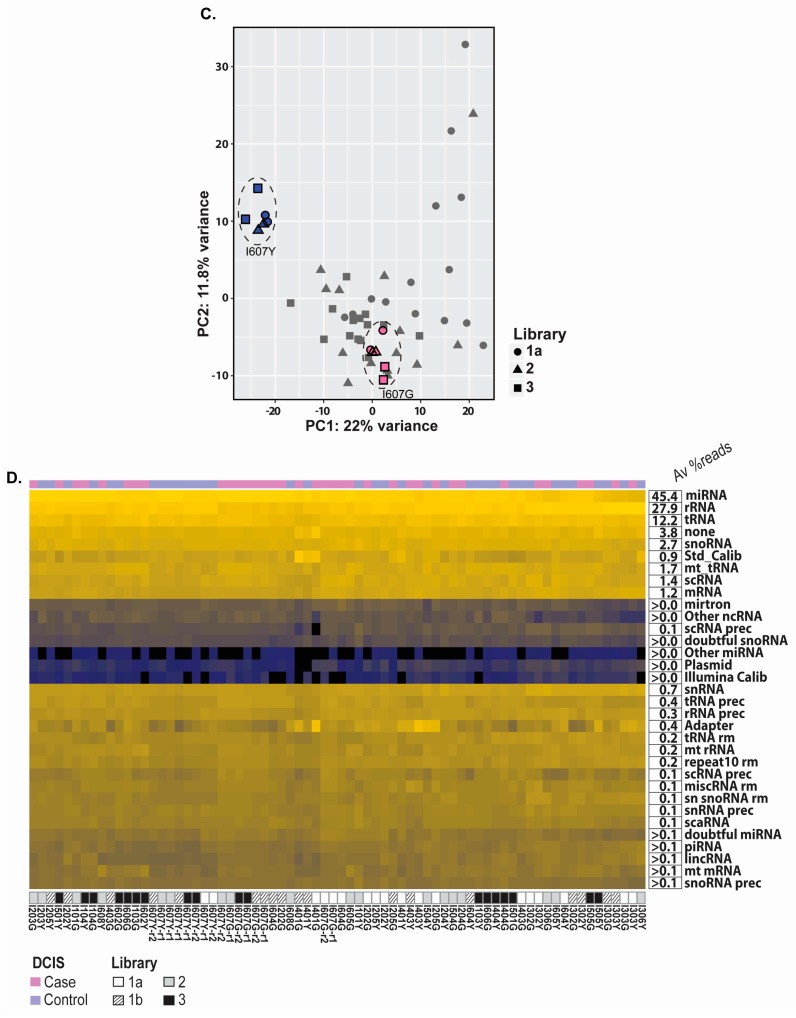
miRNA sequencing of archived DCIS specimens. (**A**) Matched case-control DCIS specimens are distributed into four libraries (black, red, green, blue ovals). Libraries 1a and 1b are duplicates. All libraries contain a control sample pair, analyzed in duplicate (I607G and I607Y). DCIS samples were obtained from six institutions: A (yellow), Kaiser Permanente Colorado (Denver, CO); B (blue), Henry Ford Health System (Detroit, MI); C (orange), Kaiser Permanente Hawaii (Honolulu, HI); D (green), Mashfield Clinic Research Foundation (Marshfield, WI): E (Brown), Kaiser Permanente Northwest (Portland, OR); F (grey), Montefiore Medical Center, Bronx, NY, New York. Libraries 1 (1a and 1b), 2 and 3 were prepared during three consecutive weeks (Weeks 1, 2 and 3); (**B**) Principal component analysis (PCA) plot of the variance between miRNA sequencing data from duplicate analyses (16 plots) in Libraries 1a and 1b. On average, each library included 575 miRNAs; (**C**) PCA plot of the variance between miRNA sequencing data from all libraries (1a (circles), 2 (triangles) and 3 (squares)). All 607Y (blue) and 607G (pink) repeats are circled with a doted line to display tight reproducibility between the different libraries; (**D**) Heat map representation of unsupervised clustering of small-RNA data obtained from 44 DCIS clinical specimens. The average distribution of all small-RNA molecules sequenced in these specimens is provided on the right. Sample ID, library position and barcoded adapters are provided below the heat map.

Using these specimens, we prepared three individual libraries, with one run in duplicate for further evaluation of the library preparation procedure (Figure 4A, Library#1a and #1b). Each of these libraries included a matched DCIS case-control sample pair (Figure 4A, I607Y and I607G; 12-year-old specimens) that was run in duplicate. The principal component analysis plot (faceted by sample) highlights the high reproducibility measured between 16 identical specimens in two distinct libraries, prepared the same week. Additionally, the PCA plot in Figure 4C shows that specimens I607Y and I607G from the three different libraries clustered well together, indicating that our optimized method is reliable for reproducing libraries containing the same FFPE RNA samples. For these libraries, known miRNAs sequenced averaged 45.4% of all small RNA reads (Figure 4D).

**Figure 5:**
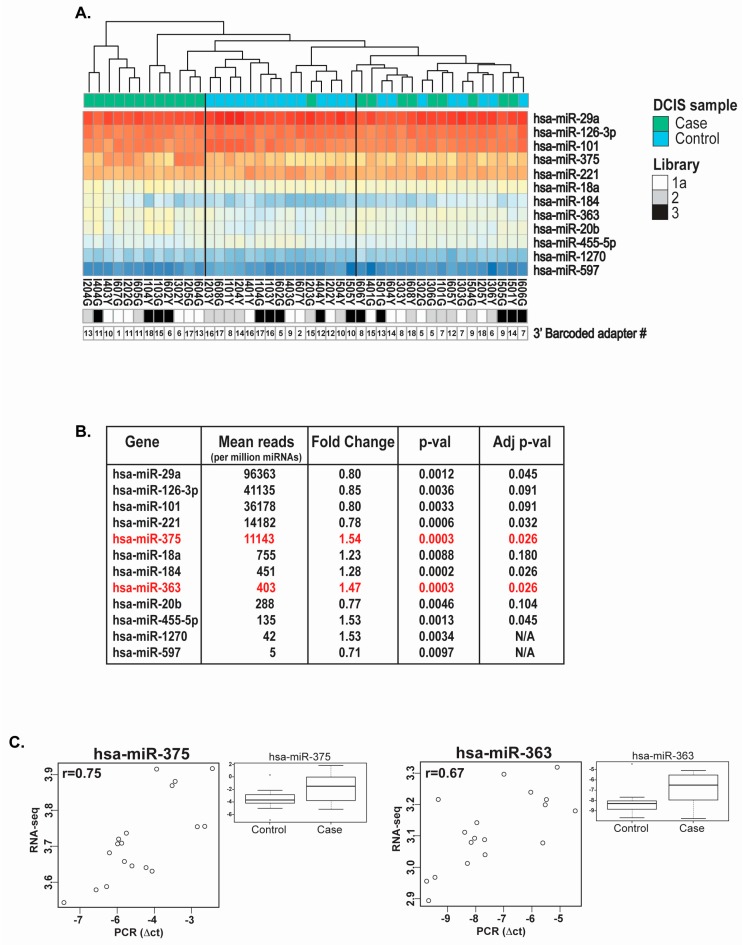
miRNAs differentially expressed in DCIS lesions based on case-control status. (**A**) Unsupervised clustering of DCIS cases (*n* = 22) and controls (*n* = 22) and heat map representation of the top 12 differentially-expressed miRNAs; (**B**) Statistical analysis on the top 12 differentially-expressed miRNAs (normalized mean of count reads (base mean), fold change difference (Log2FC), *p*-value (*p*-value) and adjusted *p*-value (Adj pval)). The two miRNAs selected for qPCR validation are highlighted in red; (**C**) Non-parametric Spearman rank correlation between quantitative PCR data of miR-375 and miR-363 in nine matched case-control DCIS specimens and miRNA sequencing data (the *r*-value represents the correlation). Two endogenous controls (mean of the comparative threshold measured for small-nucleolar RNAs (RNU44 and RNU6B) were used to normalize miRNA qPCR data. The box plot graphs display the variance stabilizing transformation (vst, DESeq2 package) for the expression of miR-375 and miR-363 in the two groups (cases and controls), with the *p*-value on differential expression between nine cases and nine controls.

The unsupervised clustering of the normalized (DESeq2 package) average read counts for the top 12 differentially-expressed miRNAs sequenced from the DCIS specimens provided three potential sample clusters: one with 12 cases, one with 14 controls and 1 case, and one with a mix of 9 cases and 8 controls. Our statistical analyses of libraries #1a, 2 and 3 initially identified a list of 20 miRNAs with significant differential expression between DCIS cases and controls (*p*-value < 0.05), of which the top 12 miRNAs (miR-29a, miR-126-3p, miR-101, miR-221, miR-375, miR-18a, miR-184, miR-363, miR-20b, miR-455-5p, miR-1270 and miR-597) are displayed in a heat map (Figure 5A). Of those top twelve differentially-expressed miRNAs, six retained statistical significance after adjustment for multiple testing (Figure 5B, miR-29a, miR-221, miR-375, miR-184, miR-363, miR-455-5p). We selected two of these miRNAs for qPCR analysis, one with a high average number of reads (above 10,000 reads per million miRNA reads), namely miR-375, and one with a low average number of reads (below 500 reads per million miRNA reads), namely miR-363, for directional validation of the differential expression identified by sequencing. Based on the remaining amounts of FFPE RNA, we selected nine DCIS case-control pairs for qPCR validation. The correlation plots for miR-375 and miR-363 provided coefficients of 0.75 and 0.67, respectively (Figure 5C) and also validated upregulation of these miRNAs in DCIS cases.

## 3. Discussion

Considerable research efforts have led to the development of molecular tools for the analysis of RNA, DNA and proteins recovered from archived FFPE tissues, with specific assays having been tailored to work with molecules compromised by extended formalin-fixation and deparaffinization [12,29,30]. We, and others, have previously demonstrated that FFPE RNA is adequate for miRNA expression profiling when using microarray/bead array and quantitative PCR technologies [14,23,31,32].

**Figure 6:**
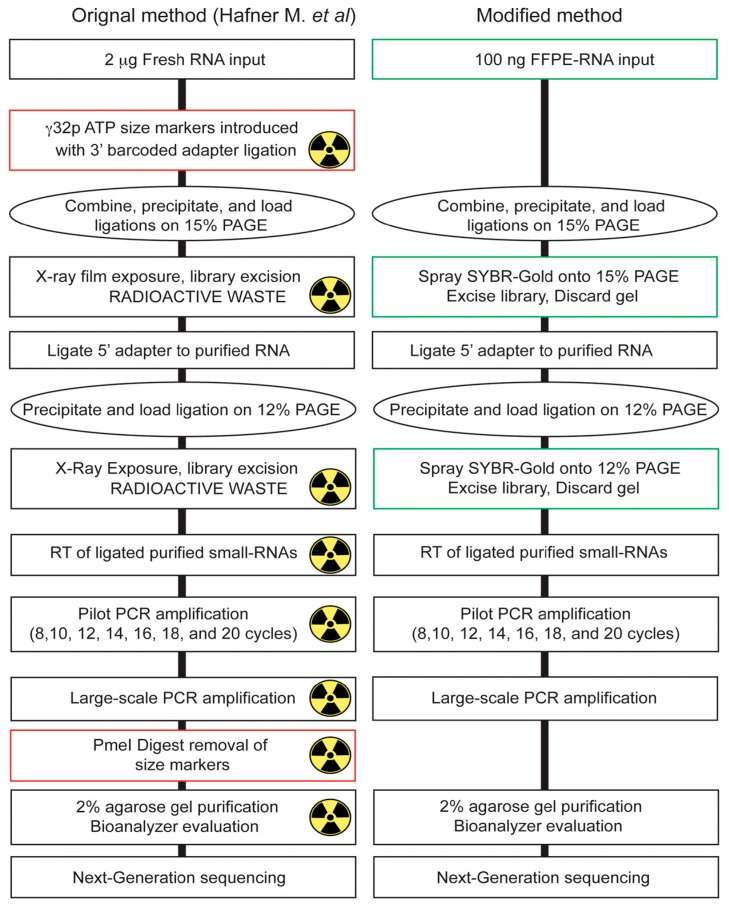
Side by side comparison of the original and modified cDNA library preparation method. Molecular biology steps used for small-RNA cDNA library preparation. The green boxes display changes applied to the original method, whereas red boxes display steps that were removed. The radioactive sign in the original method indicates steps where γ−32ATP-labeled size markers were present.

In this study, we evaluated and optimized a laboratory-based 3′ barcoding cDNA library preparation protocol for next-generation sequencing (NGS) of small-RNAs recovered from archived FFPE specimens, based on the protocol developed by Hafner et al. [24]. We selected this approach as it displayed four major advantages for preparing small-RNA cDNA libraries with FFPE-RNA: First, the key feature of this protocol for working with limited amounts of FFPE RNA was that all libraries (i.e., 18) are pooled together after initial ligation of the 18 unique 3′ barcoded adapters (to 18 unique RNA samples), which allows the reduction of RNA input from each sample, due to a carrier effect that minimizes subsequent material losses during gel purifications and precipitations; second, all experimental steps of this laboratory-based protocol, in contrast to commercial kit-based methods, can be optimized, as reagents and buffers are prepared in the laboratory and all biochemical reactions are performed with widely available commercial enzymes; third, the preparation of a pilot PCR step allows optimal identification of the number of PCR cycles necessary for the amplification of ligated and barcoded small-RNAs from a small amount of FFPE RNA material; fourth, the separation on polyacrylamide gels (PAGE) allows specific size selection, excision and amplification of different small RNA populations (i.e., miRNAs, piRNAs, etc.), even when specifically purifying miRNAs (Figure S1). Nevertheless, there were two aspects of the procedure developed by Hafner et al. [24] that we modified and optimized in order to allow its application in any molecular laboratory, but also to improve its performance when working with FFPE-RNA (Figure 6). First, in the method developed by Hafner et al. [24], γ−32ATP-labeled size markers were introduced in the initial ligation for subsequent PAGE selection, which represented a major inconvenience due to the production of radioactive waste, requiring proper permit application, disposal, as well as immobilization of equipment due to potential radioactive contaminations. With our optimized protocol, we synthesized RNA size markers (Figure S2) containing a unique 3′ barcoded adapter (for evaluation of contamination), which underwent ligation of the 5′ adapter and which were run in wells adjacent to the small-RNA libraries (see Figure 1. 15% PAGE). By using SYBR^®^ Gold staining (spraying) of the gel, we visually evaluated and excised a portion of the gel corresponding to the small-RNA population of interest. Secondly, due to the introduction and amplification of γ−32ATP-labeled size markers with the libraries during the pilot PCR reaction, which affected the output due to lower RNA input and quality, the digestion by the restriction enzyme PmeI to remove these radio-labeled primers was omitted. By introducing an additional barcode on the size markers, we were able to evaluate the low read contribution (<1000 total reads on average).

We evaluated the performance of our modified approach using gold standard matched fresh/frozen and FFPE human tissues/cells and demonstrated that FFPE RNA provides high quality miRNA expression data relative to its matched fresh/frozen counterpart, when using NGS [19,20,21,22,33]. By examining assay repeatability on decreasing amounts of FFPE RNA, we demonstrated that 100 ng of FFPE RNA were sufficient for sequencing miRNAs from archived specimens. We also found that our modified procedure was highly reproducible (*r* > 0.96) when using specimens up to 35 years old, further suggesting that archived specimens subjected to extended storage contain well-preserved miRNAs that can be purified, cloned and sequenced [12,19,20,21,22,29,33]. Our repeated measures performed at different time intervals further display that this laboratory-based optimized protocol should be applicable to large-scale FFPE-RNA studies, which require successive weeks of experimentation. In our experience, we have found that this laboratory-based method is extremely cost effective, when compared to commercial kits. In fact, after initial purchase, aliquoting (2.5 μL per tube) and storage of the 3′ barcoded adapters at −80 °C, we determined that these would allow preparation of at least 145 libraries each containing 18 samples (~2600 samples) over a period of two years (optimal condition for stored 3′ barcoded adapters). Our overall cost estimates, including initial purchase of eighteen 3′ barcoded adapters, primers, size markers, enzymes, reagents and consumables suggested an average cost of $40 for the preparation of a single small-RNA cDNA library, when working with a sample set of 650 specimens (i.e., one forth of 2600 samples) or approximately 36 libraries of 18 specimens. This cost could be further decreased if more samples were to be analyzed. Although commercial kits for the preparation of small-RNA libraries vary in cost, the most affordable ones can be evaluated around $100 per sample, which is more than twice the cost of our sample preparation.

In our final analyses, we evaluated our modified procedure by applying it to archived clinical breast ductal carcinoma in situ (DCIS) specimens, a type of non-invasive lesion characterized by an intraductal neoplastic proliferation of epithelial cells, considered to be a non-obligate precursor of IBC, but associated with an increased risk of invasive breast cancer (IBC) development; indeed, 5%–14% of all patients diagnosed with DCIS and treated with breast conserving therapy with or without radiation experience an ipsilateral recurrence within 10 years [34,35]. It has been suggested that the analysis of DCIS lesions may provide insights into the molecular changes associated with risk of IBC development, hence the miRNA expression analysis of these lesions [1]. However, given the latent interval between detection of DCIS lesions and the development of subsequent IBC, as well as the time required for prospectively collecting DCIS specimens, the use of preserved specimens is almost inevitable. To date, only one cross-sectional qPCR-based miRNA expression study has been performed on archived DCIS specimens, which indicated that DCIS lesions from patients (*n* = 8) who already have invasive breast cancer display miRNA expression differences, when compared to miRNA expression in normal breast tissue (*n* = 8) [36]. To extend work in this area, in a prospective setting, we chose to evaluate our optimized procedure by conducting a small case-control study nested in a cohort of DCIS specimens collected from six different research centers across the Unites States and between which fixation protocols were not standardized. The 44 selected specimens included 22 DCIS cases, in which the patient subsequently developed IBC, and 22 DCIS controls matched 1:1 to the corresponding case and who did not develop IBC within the same follow-up interval as that for the matched case. Our measures provided highly reproducible data between repeats, for specimens stored for up to 26 years from the different research institutions, with an average of 575 miRNAs detected and analyzed. Our sequencing analyses revealed that six miRNAs retained significance after adjustment for multiple testing, namely miR-29a, miR-221, miR-375, miR-184, miR-363 and miR-455-5p, of which two miRNAs, namely miR-375 and miR-363, were further validated by qPCR. It is interesting to note that in a previous miRNA expression study, on breast lobular carcinoma in situ (LCIS) lesions (another type of non-invasive neoplastic breast cellular proliferation), we had identified similar miRNA expression changes between LCIS lesions and synchronous IBCs, which included expression upregulation of miR-375. Lentiviral expression of miR-375 in non-tumorigenic breast epithelial MCF10A cells in 3D culture, in vitro, induced loss of cellular polarity, suggesting that deregulation of this miRNA may play important roles in neoplastic lesions, with regard to breast cancer progression [32]. The identification of miRNA markers associated with risk of invasive breast cancer development may play an important role in improving preventive patient care.

Our analyses indicate that our optimized laboratory-based barcoded cDNA library preparation represents a valuable tool for the analysis of archived FFPE tissues from different eras and from different institutions and suggests that archived DCIS specimens may contain molecular markers associated with risk of IBC development.

## 4. Materials and Methods

### 4.1. Cell Lines and Tissues

Human non-tumorigenic MCF10A cells (ATCC, Cat No. CRL-10317) were cultured in DMEM/F12 medium supplemented with 5% horse serum, 20 ng/mL EGF, 0.5 μg/mL hydrocortisone, 100 ng/mL cholera toxin, 10 mg/mL insulin, 50 u/mL penicillin and 50 μg/mL streptomycin (media changed every two days) at 95% air and 5% CO_2_. MCF7 cells (ATCC, Cat No. HTB-22D) were cultured in DMEM with 2 mmol/L l-glutamine and 10% fetal bovine serum (media changed every two days) at 95% air and 5% CO_2_ and were grown separately in nine 100-mm Petri dishes until reaching 60% confluence. The cells were washed (1× PBS pH 7.4) and gently scraped from the Petri dishes and collected in 50-mL Corning tubes, where they were spun at 800× *g* for 5 min at 4 °C. Control fresh RNA (Bioanalyzer RIN = 10) was obtained from a subset of cells using TRIzol. The rest of the cell pellet was placed into 15 mL of 3.7% buffered formaldehyde (2 mL 27% formaldehyde, 2 mL 10× PBS pH 7.4 and 16 mL nuclease-free water from Ambion, Austin, TX, USA) for 4 h at room temperature, and FFPE blocks were prepared at the histopathology facility at the Albert Einstein College of Medicine. Ten-micron sections were cut, and RNA was extracted following the procedure detailed below. Human normal and tumor tissues were obtained from the Montefiore Medical Center (MMC), Bronx, NY, USA, after submission and approval of a study proposal by the Institutional Review Board (IRB). For the purpose of this study, the specimens were obtained under two IRBs, which included IRB#2010-366 approved on 27 July 2010, and IRB#2014-3611 approved on 24 June 2014. IRBs were maintained throughout the study and active on 10 March 2017. No animal specimens were analyzed in this study.

### 4.2. DCIS Specimens and Tissue Collection

Fully-anonymized FFPE human breast ductal carcinoma in situ (DCIS) specimens were gathered from 6 health centers for our miRNA sequencing study. The six providing centers were Kaiser Permanente Colorado (Denver, CO, USA), Henry Ford Health System (Detroit, MI, USA), Kaiser Permanente Hawaii (Honolulu, HI, USA), Marshfield Clinic Research Foundation (Marshfield, WI, USA), Kaiser Permanente Northwest (Portland, OR, USA) and Montefiore Medical Center (Bronx, NY, USA). We established a case-control study of breast cancer nested in a cohort of women with ductal carcinoma in situ (DCIS) of the breast, where cases (*n* = 22) were women who developed subsequent invasive breast cancer (>6 months after DCIS diagnosis) and controls (*n* = 22) were women with DCIS who did not develop breast cancer within the same time interval, without history of a breast cancer. Cases and controls were matched based on age of DCIS diagnosis. For these samples, each individual center received approval from their IRBs. For each block, 5-μm tissue sections were cut and stained with hematoxylin and eosin (H&E) for pathological review. DCIS lesions were identified by microscopy and circled on the cover slips, and these H&E slides were then used as tissue guides for macro-dissection of the DCIS lesions. For each individual FFPE DCIS tissue block, a total of ten 10-μm sections was macrodissected prior to RNA extraction in siliconized nuclease-free Eppendorf tubes [32]. RNA extraction was performed on matched case-control specimens, the same day as tissue macrodissection, to prevent tissue desiccation, RNA damage and technical differences.

### 4.3. RNA Extraction

RNA extraction from fresh cells (homogenized in tubes by pipetting up and down) and frozen tissues (ground with a Tissue Tearor (Biospec Products, Inc., Bartlesville, OK, USA) in 5-mL glass tubes) was performed using TRIzol and following the manufacturer’s instructions. RNA extraction from FFPE tissues was performed following our optimized simultaneous RNA/DNA extraction [23,37]. Briefly, the FFPE tissues were de-paraffinized using Citri-Solv (Thermo Fisher Scientific, Inc., Watham, MA, USA) at room temperature on a Thermomixer (Eppendorf, Hamburg, Germany), followed by ethanol washes on ice, a 1× PBS wash and re-hydration in the presence of RNase-inhibitors. The macro-dissected tissues were digested with fresh proteinase K (3 mg/mL), at 59 °C for 1 h. The digested tissues underwent butanol-1 extraction to reach a final volume of 100 μL. This solution was homogenized in 1 mL of TRIzol (Invitrogen, Grand Island, NY, USA) following the manufacturer’s instructions. For RNA recovery, the upper phase of the TRIzol was transferred to a new siliconized Eppendorf and precipitated with 0.1 mg/mL linear acrylamide (1 μL), 3 M sodium acetate (18 μL) and 600 μL of isopropanol. The tubes were stored at −20 °C overnight and spun at 14,000 rpm for 30 min at 4 °C. The precipitated RNA pellet was washed with 200 μL of 70% RNase-free ethanol, dried and re-suspended in 12 μL of RNase-free 1× Tris/EDTA solution and incubated for 30 min at 70 °C. The RNA was quantified on a total RNA chip on a Bioanalyzer (Agilent, Danbury, CT, USA). The lower (organic) phase of TRIzol, which contained genomic DNA, was further processed following the procedure described in Kotorashvili et al. [23].

### 4.4. Optimized Small-RNA cDNA Library Preparation for Illumina HiSeq-2500 Sequencing

Small-RNA sequencing of FFPE RNA was optimized (100 ng) using the cDNA library preparation protocol described by Hafner et al. [24] with modifications detailed below. We used 18 barcoded 3′ adapters for library preparation of 18 individual samples, per run (Figure S2). For ligation of the 3′ barcoded adapters, we made the 10× RNA ligase buffer (without ATP), which contained 343 μL Ambion™ nuclease-free water (not DEPC-treated) (AM9932, Thermo Fisher Scientific), 500 μL UltraPure™ 1M Tris-HCl Buffer pH 7.5 (15567027, Thermo Fisher Scientific), 100 μL Ambion™ 1M MgCl_2_ (AM9530G, Thermo Fisher Scientific), 50 μL 20 mg/mL BSA, acetylated (B8894, Sigma-Aldrich, St. Louis, MO, USA) and 7 μL 14 M 2-mercaptoethanol (O3446I, Thermo Fisher Scientific). Our master mix RNA ligation reaction was set up for 18 individual ligations and contained 40 μL 10× RNA ligase buffer (without ATP), 120 μL 50% aqueous Dimethyl sulfoxide (DMSO, D9170, Sigma-Aldrich, St. Louis, MO, USA) and 10 μL calibrator cocktail (0.026 nM). Each of the 18 individual ligation reactions was set up by adding 9.5 μL RNA (100 ng), 8.5 μL of the master mix, 1 μL of 50 μM adenylated barcoded 3′ adapter (custom order, Integrated DNA Technologies, Coralville, IA, USA) and heating them to 90 °C for 1 min and back on ice prior to adding 1 μL of the 0.5× diluted T4 RNA Ligase 2, truncated K227Q (M0351L, New England Biolabs, Ipswich, MA, USA) and incubating the 18 reactions on ice overnight (O/N), in a 4 °C room. After deactivation of the enzyme at 90 °C for 1 min and the addition of 1.2 μL of Glycoblue mix (1 μL Glycoblue™ Coprecipitant (15 mg/mL) (AM9516, Thermo Fisher Scientific) in 26 μL NaCl (5M) (AM9579, Thermo Fisher Scientific)) and 63 μL 100% ethanol to each tube, all 18 reactions were pooled in a Fisherbrand™ siliconized low-retention microcentrifuge tube (02-681-331, Thermo Fisher Scientific) and incubated on ice for 1 h after gentle mixing. The tube was spun down at 14,000 rpm at 4 °C for 1 h, the pellet air dried and resuspended in 20 μL nuclease-free water and 20 μL denaturing polyacrylamid (PAA) gel loading solution (14.25 mL deionized formamide (AM9342, Thermo Fisher Scientific), 150 μL 0.5 M sodium EDTA, pH 8.0 (E5134, Sigma-Aldrich), 15 mg Bromophenol Blue (B0126, SigmaAldrich, St. Louis, MO, USA) and 600 μL nuclease-free water), prior to loading on a 15% polyacrylamide gel (PAGE) (9 mL Urea System Diluent (EC-840, National Diagnostics, Atlanta, GA, USA), 18 mL Urea System Concentrate (EC-830, National Diagnostics), 3 mL Urea System Buffer (EC-835, National Diagnostics), 240 μL 9% ammonium persulfate (327081000, Thermo Fisher Scientific) and 12 μL tetramethylethylenediamine (TEMED) (138450500, Thermo Fisher Scientific)). Rather than using radioactivity for size selection, we used the 19-nt and 24-nt (plus the size of the 3′ adapter or total size of 39 and 45 nt, respectively) RNA molecules (Integrated DNA Technologies, Coralville, IA, USA) each run in separate wells and at least 2 wells away from the libraries, on each side. The acrylamide gel was run in 0.5× TBE solution (15581044, Thermo Fisher Scientific) at 450 volts for 90 min for adequate separation. We then opened the glass holding the gel and exposed it to SYBR^®^ Gold™ dye (S11494, Thermo Fisher Scientific) (10 μL SYBR Gold in 50 mL 0.5× Tris-Borate EDTA (TBE)) by spraying the gel, prior to excising the size-selection and excision of the library on a Safe Imager^TM^ 2.0 (Invitrogen). The excised gel piece was subjected to a gel breaker (3388-100, IST Engineering, Milpitas, CA, USA), and 300 μL NaCl (400 mM) were added to it and the tube incubated on a thermomixer at 1100 rpm at 4 °C, overnight. Ligated RNA size marker oligos (with 5′ adapter, also run on the gel) were excised separately and similarly precipitated overnight. The next day, the eluted RNA samples were filtered, precipitated for 1 h on ice by the addition of 900 μL of 100% ethanol and pelleted at 14,000 rpm for 1 h at 4 °C. The small-RNA pellet (RNA libraries) was resuspended in 9 μL of water to which 2 μL of 10× RNA ligase buffer with ATP [24], 6 μL 50% aqueous DMSO and 1 μL 5′ adapter, incubated at 90 °C for 1 min and on ice for 2 min, before adding 2 μL T4 RNA ligase 1 and incubating it at 37 °C for 1 h were added. After 5′ adapter ligation, the addition of 20 μL of PAA gel loading solution, the library and size markers were loaded onto a 12% PAGE (12.6 mL Urea System Diluent, 14.4 mL Urea System Concentrate, 3 mL Urea System Buffer, 240 μL 9% ammonium persulfate, and 12 μL TEMED) and run for 90 min at 450 volts in 0.5× TBE. The gel was cooled down, sprayed with SYBR^®^ Gold™, and the size-selected libraries were excised and subjected to a gel breaker and incubated in 300 μL NaCl (300 mM) and 1 μL 100 μM 3′ reverse transcription (RT) primer (Figure S1) on a thermomixer at 1100 rpm at 4 °C O/N. The next day, the filtered solution was precipitated by the addition of 900 μL 100% ethanol, incubation on ice for 1 h and spun for 1 h at 14,000 rpm, at 4 °C. The RNA pellet was resuspended in 5.6 μL nuclease-free water, and the RT reaction (15 μL final volume) was set up (3 μL 5× first strand buffer, 1.5 μL DTT 0.1 M and 4.2 μL dNTP Mix (2 mM each)), heated at 90 °C for 30 s, transferred to a 50 °C heat-block for 2 min prior to the addition of 0.75 μL SuperScript^®^ III Reverse Transcriptase (18080-093, Thermo Fisher Scientific), incubated for 30 min at 50 °C and deactivated at 95 °C for 1 min prior to the addition of 95 μL of nuclease-free water, to reach a final volume of 110 μL cDNA solution. A pilot PCR reaction was set up by adding 10 μL 10× PCR buffer, 10 μL dNTP Mix (2 mM each), 10 μL cDNA, 67 μL nuclease-free water, 0.5 μL 3′ PCR primer, 0.5 μL 5′ PCR primer and 2 μL Titanium^®^ Taq DNA Polymerase (639208, Clontech Laboratories, Mountain View, CA, USA) and subjected to 8, 12, 14, 16, 18 and 20 cycles, at which 10-μL aliquots were subsequently collected, prior to 2% agarose gel analysis for identification of the optimal PCR amplification cycle. Six large-scale PCR reactions (same reagents as pilot PCR), each containing 10 μL cDNA subjected to the optimal PCR cycle amplification, were run, combined and precipitated by adding 30 μL NaCl (5M) and 950 μL 100% ethanol by incubation at −20 °C O/N. PCR DNA pellets were obtained at 14,000 rpm for 1 h at 4 °C, vacuum-dried, resuspended, and the PCR products were separated on a 2% agarose gel containing 5 μL ethidium bromide; and the 100-nt library band was excised (upper band), purified using the QIAquick Gel Extraction Kit (28704, Qiagen, Hilden, North Rhine-Westphalia, Germany) and quantified using the Qubit^®^ dsDNA HS Assay Kit (Q32854, Thermo Fisher Scientific). The cDNA libraries were then sequenced (single-read 50 cycles) on a HiSeq 2500 Sequencing System (SY-401-2501, Illumina, San Diego, CA, USA). After sequencing, the FASTQ files containing the sequencing raw data were processed at Rockefeller University, New York, NY, USA, using the RNAworld pipeline (the laboratory of Thomas Tuschl) [38]. The count tables were then subjected to statistical analyses.

### 4.5. Quantitative PCR

Four microRNAs, which displayed significant differential expression between DCIS cases and matched control specimens, were selected for quantitative PCR (qPCR) validation using total RNA from 9 randomly-selected DCIS case-control sample pairs that had been used for the sequencing experiments. Taqman^®^ miRNA primer assays for miR-221, miR-375, miR-363 and miR-20b were obtained and qPCR experiments were performed using Taqman^®^ microRNA reverse-transcription kits and Taqman^®^ microRNA master mix PCR kits following the manufacturer’s instructions (Thermo Scientific Fisher, CA, USA) and also described in Giricz et al. [32]. Quantitative PCR measurements were performed on a Step-One-Plus instrument using the manufacturer’s recommended 96-well plates and covers. Quantitative data were transferred to Excel for statistical analysis. We used RNU44 and RNU6b as endogenous controls for data normalization. Relative quantification of the gene transcripts (Δ*C*_t_) was obtained by subtracting the mean comparative threshold (*C*_t_) of the endogenous controls (RNU44 and RNU6b) from the mean *C*_t_ value of the miRNA of interest.

### 4.6. Statistical Analyses

Raw FASTQ data files obtained from a HiSeq 2500 sequencer at the Epigenomics and Genomics Shared facility at the Albert Einstein College of Medicine were imported into the RNAworld private server at Rockefeller University, where adapter trimming and microRNA read identification was performed and exported to text files. All statistical analyses were performed using dedicated Bioconductor packages in the R platform as detailed below. For the optimization process where fresh-frozen data and FFPE data were compared, we calculated and compared the respective log_2_ frequencies of upper quartile-normalized miRNA counts (“edgeR” package [28]). Heat maps (Figure 3 and Figure 5) were generated using the “pheatmap” and “NMF” Bioconductor packages, applying the indicated distance metrics and clustering methods. For miRNA expression and data reduction analyses performed on the DCIS data (Figure 4 and Figure 5), we corrected for batch effect using ComBat (“sva” package [39]) and then applied a DESeq2 model on aggregated miRNA counts (i.e., summed technical repeats). For PCA plots, we used variance-stabilizing transformations (“vst”) of miRNA counts (“DESeq2” package [40]). For quantitative PCR analyses, miRNA reads obtained by sequencing after “vst” transformation were compared to the qPCR Delta Delta Comparative Threshold (ΔΔ*C*_t_) data. Annotated R scripts are available upon request.

## 5. Conclusions

Our application of the cDNA library preparation protocol, developed by Hafner et al. [24] for sequencing of small RNAs, demonstrates that miRNAs from archived specimens are well preserved and can be robustly analyzed using next-generation sequencing technology. By pooling barcoded small-RNAs from FFPE specimens after initial ligation of the 3′ barcode, this unique approach allows to work with smaller amounts of material, a valuable feature when handling degraded material. The application of this approach to the retrospective analysis of clinical specimens may provide valuable data on miRNA expression changes associated with cancer risk and progression.

## Figures and Tables

**Table 1:** Archived breast ductal carcinoma in situ (DCIS) specimens. DCIS specimen information includes case-control status, age of blocks, age of patients and time to breast cancer development. Cases and controls were matched on age at diagnosis, and controls were individuals who did not develop breast cancer within the same time interval.

Cases	Tissue Age (Year)	* Race Ethnic	Age at DCIS	** Time to BC (Months)	Controls	Tissue Age (Year)	* Race Ethnic	Age at DCIS
I101G	9	W/NH	62	80	I101Y	9	W/NH	62
I103G	8	W/NH	64	8	I103Y	8	W/NH	64
I104Y	7	W/H	79	12	I104G	6	W/H	78
I202G	8	B/NH	84	16	I202Y	8	W/NH	83
I203G	8	B/NH	50	78	I203Y	8	W/NH	50
I204G	13	B/NH	47	66	I204Y	13	W/NH	47
I205G	10	B/NH	68	74	I205Y	10	U/NH	68
I302Y	18	U/NH	62	82	I302G	17	U/NH	64
I303Y	26	W/NH	56	260	I303G	25	U/NH	56
I306G	22	U/NH	49	13	I306Y	24	A/NH	45
I401G	3	W/NH	77	10	I401Y	3	W/NH	75
I403Y	12	W/NH	42	35	I403G	12	W/NH	43
I404G	9	W/NH	76	59	I404Y	10	W/NH	75
I501Y	14	A/NH	46	99	I501G	15	W/NH	45
I504G	22	W/NH	48	168	I504Y	22	W/NH	47
I505G	28	W/NH	41	97	I505Y	28	W/NH	41
I602Y	6	B/NH	70	45	I602G	7	W/NH	70
I604G	11	W/NH	83	8	I604Y	12	W/NH	83
I605G	12	W/NH	74	31	I605Y	12	W/H	75
I606Y	12	B/NH	67	57	I606G	12	W/NH	67
I607G	11	B/NH	78	99	I607Y	12	W/H	78
I608Y	6	B/NH	41	49	I608G	7	U/NH	41

* Race is defined by the following lettering: W for white/caucasian, B for Black/African-American, A for Asian, U for unknown, Ethnic background is defined by H for Hispanic/Latino or NH for Non-Hispanic/Latino. ** Time between diagnosis of DCIS and Breast cancer (BC) in years.

## Supplementary Materials

Supplementary materials can be found at www.mdpi.com/1422-0067/18/3/627/s1.
