# Supplementary material for: Evaluation and Adaptation of a Laboratory-Based cDNA Library Preparation Protocol for Retrospective Sequencing of Archived MicroRNAs from up to 35-Year-Old Clinical FFPE Specimens
Source: Int J Mol Sci. 2017 Mar 14;18(3):627. doi: 10.3390/ijms18030627 (PMC5372640; doi:10.3390/ijms18030627)
Supplement: Supplementary file 1 [file ijms-18-00627-s001.pdf]

Supplementary Materials: Evaluation and Adaptation of a Laboratory-Based cDNA Library Preparation Protocol for Retrospective Sequencing of Archived MicroRNAs from up to 35-Year-Old Clinical FFPE Specimens

Olivier Loudig, Tao Wang, Kenny Ye, Juan Lin, Yihong Wang, Andrew Ramnauth, Christina Liu, Azadeh Stark, Dhananjay Chitale, Robert Greenlee, Deborah Multerer, Stacey Honda, Yihe Daida, Heather Spencer Feigelson, Andrew Glass, Fergus J. Couch, Thomas Rohan and Iddo Z. Ben-Dov

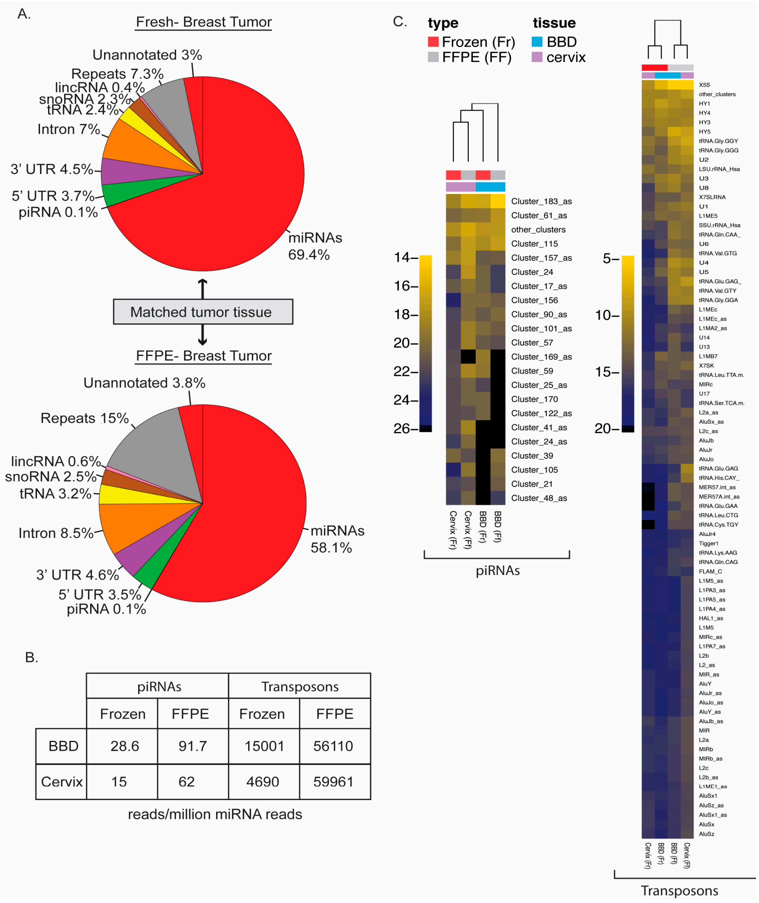

Figure S1. Evaluation of piwiRNAs and transposons sequencing reads. In parallel to the automated processing used by the Rockefeller small RNA pipeline, FASTQ files underwent demultiplexing and adapter trimming using cutadapt [25]. We then applied the piPipes small RNA annotation pipeline to detect and quantify piRNAs and transposable elements in the small RNA libraries initially designed for miRNA sequencing [26]. (**A**) Pie charts display the main small RNA categories identified by piPipes, in matched fresh frozen and FFPE breast tumor specimens. Based on these samples, piRNAs constitute less than 0.1% of the small RNA reads; (**B**) Total piRNA and transposon read counts normalized as reads per million miRNA read counts in matched frozen and FFPE breast and cervix specimens (relative enrichment in FFPE is likely explained by greater loss of miRNA molecules during preparation of these specimens); (**C**) Heat maps and hierarchical clustering generated with the Bioconductor package "NMF" [27] from read counts, which were normalized using edgeR [28]. Only the top piRNA clusters and transposons are shown, and brighter shades represent higher abundance.

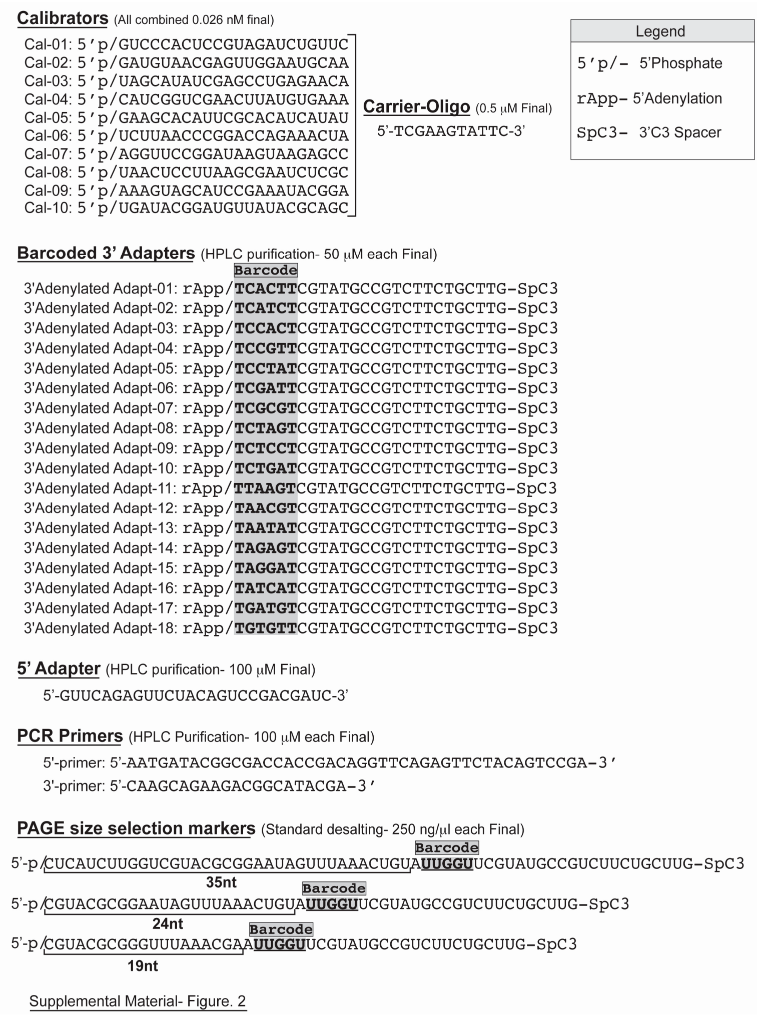

Figure S2. Adapters and primers. Ten calibrator oligonucleotides at a final concentration of 0.026 nM resuspended in a RNAse-free solution containing 0.5 mM carrier oligonucleotide. Eighteen 3′ barcoded adapters adenylated in 5′ (rApp) and containing a spacer in 3′ (SpC3) were used at a 50 mM final concentration. One 5′ adapter compatible with Illumina HiSeq2500 sequencer was used at a 100mM final solution. Three size marker oligonucleotides were used in pair for miRNA size selection (19 and 24 nt oligonucleotides) or for piwiRNA size selection (24 and 35 nt oligonucleotides) on a polyacrilamide gel, at a concentration of 250 ng/mL. All three size marker oligonucleotides contain a unique barcode for sequencing trimming.
